# The protective effect of melatonin supplementation against taxol-induced testicular cytotoxicity in adult rats

**DOI:** 10.1590/1414-431X2021e11614

**Published:** 2022-02-04

**Authors:** H.R. Aboelwafa, R.A. Ramadan, A.F. El-Kott, F.M. Abdelhamid

**Affiliations:** 1 Department of Biological and Geological Sciences, Faculty of Education, Ain Shams University, Cairo, Egypt; 2 Biology Department, Faculty of Science, King Khalid University, Abha, Saudi Arabia; 3 Zoology Department, College of Science, Damanhour University, Damanhour, Egypt

**Keywords:** Chemotherapeutic drugs, Testes, Melatonin, Histology, Immunohistochemistry, Ultrastructure

## Abstract

The aim of the present investigation was to study the toxic influences of taxol (TXL) on the testes of rats and the protective impact of melatonin (MLT) against such effects. Rats were classified into control, sham, TXL, MLT, and MLT+TXL-treated groups. Histological and ultrastructural changes were observed in testicular tissues of TXL-intoxicated rats including thickening of tunica albuginea and degenerative alterations in spermatogenic, Sertoli, and Leydig cells. A significant increase (P≤0.05) was found in the thickness of tunica albuginea and numbers of tubules without sperm, apoptotic germinal epithelia, and apoptotic Leydig cells, whereas the diameter of tubules and height of germinal epithelia displayed a significant decrease (P≤0.05) compared with the control, sham, and MLT-treated groups. Immunohistochemically, a marked decrease (P≤0.05) in Bcl-2 immunoreactivity and significant elevation (P≤0.05) in P53 and caspase-3 immunoreactivities were recorded. Co-treatment of MLT and TXL modulated such histological, histomorphometrical, and ultrastructural changes induced by TXL. Also, MLT had a protective effect against testicular apoptosis induced by TXL, as shown by the elevated expression of Bcl-2 and decreased expression of P53 and caspase-3. In conclusion, the current investigation proved that MLT had a protective role against TXL-induced testicular cytotoxicity, which may be a result of inhibition of testicular apoptosis.

## Introduction

Cancer is a serious public health problem worldwide and a leading cause of death, claiming the lives of nearly 18.1 million individuals each year (1). The worldwide burden of cancer is predicted to inevitably increase, primarily as a result of global population growth and aging, as well as increasing cancer-related lifestyles, especially smoking, unhealthy diets, and lack of physical activity. The economic impact of cancer, including heavy financial investments and premature deaths, is serious and is expected to increase substantially in the future. Chemotherapy is very effective in slowing the progression of cancer (2), but unfortunately, many anti-cancer drugs have deleterious side effects, including on the reproductive system. Particularly, chemotherapy can change hormone levels and sperm quality, resulting in reduced fertility or infertility (3).

As an efficient chemotherapeutic agent and mitotic inhibitor, taxol (TXL), the first authorized paclitaxel formulation in 1992, is arguably one of the most effective anti-cancer drugs of all times, although some other formulations are available. It has been successfully used to treat various types of solid tumors including non-small cell lung, thyroid, stomach, colon, breast, ovarian, prostate, bladder, and head and neck cancers (4). TXL, a plant alkaloid and an antimicrotubule agent, promotes tubulin polymerization and prevents microtubule depolymerization, which is crucial for cell division. Furthermore, other mechanisms such as intracellular signaling alteration and organelle transportation have a vital role in the anti-cancer properties of TXL (5). Meanwhile, TXL does not have a targeted action like other chemotherapeutic drugs, and it causes cell death, leading to severe side effects that reduce its efficiency (6).

Despite the disadvantages and consequences of TXL chemotherapy, it remains the first choice and most commonly used strategy in cancer therapy. Worldwide, researchers are exploring approaches to reduce the side effects of such chemotherapeutic drugs to enhance the vitality of patients, reduce pain, and increase their lifespan. Therefore, researchers are very interested in mixing anti-cancer drugs with natural products to maximize their effectiveness and reduce their side effects.

Melatonin (MLT) is a natural derivative of the amino acid tryptophan (N-acetyl-5-methoxy tryptamine), which was primarily extracted from the bovine pineal gland. MLT was previously thought to be produced exclusively in the pineal glands and to function like a hormone controlling sleep and circadian rhythm (7). Recently, MLT-related enzymes were discovered in numerous other tissues and MLT receptors were found in many cells (8). The combination of these facts and the discovery of the powerful antioxidant, free-radical scavenging, and cell-modulating properties of MLT strengthen the status of MLT as a brain-limited hormone to a ubiquitous substance involved in the regulation of a wide range of biological activities, including energy balance, seasonal breeding, cardiovascular, neuroendocrine, and immunological functions, as well as anti-aging and anti-ischemic properties (9). MLT has been shown to inhibit the progression of tumor cell growth in various types of cancers (10). In parallel, many scientists have focused their research on the potential advantages of MLT to curb the side effects of chemotherapy drugs.

The testis is considered the most important organ of the male reproductive system. It has two main functions: the synthesis of steroid hormones and the production of sperm. Unfortunately, it is considered the most sensitive target organ in the body for injury from many drugs, environmental toxic agents, and other hazardous factors because its germinal epithelium divides rapidly during mitosis and meiosis (11). Accordingly, the present investigation was planned to assess the deleterious effects of TXL on the testicular tissues of adult rats and the probable protective effect of MLT supplementation against those effects using histological, histomorphometrical, immunohistochemical, and ultrastructural approaches.

## Material and Methods

### Experimental animals

Fifty adult male Wistar rats (*Rattus norvegicus*) weighing 250-300 g and 16-18 weeks old were obtained from the closed colony of the Theodor Bilharz Research Institute, El-Giza, Egypt. Animals were kept in clean plastic crates lined with wood shavings and were fed a standard rodent pellet diet, water, and milk *ad libitum*. The animals were kept under suitable hygienic circumstances, in 12-h light/dark periods, 25°C temperature, and 55±5% relative humidity. All rats were allowed to adapt for two weeks prior to experimentation. The current investigation was performed in accordance with the guidelines for animal research approved by the Institutional Animal Ethics Committee of Ain Shams University.

### Pharmacological materials

TXL is produced by Bristol-Myers Squibb Company (USA, product number NJ 08543) in the form of a viscous, transparent, colorless to slightly yellow solution. MLT was obtained in powder form from Sigma-Aldrich Chemie GmbH (Germany, product number Q20D023). The other chemicals used in the present research were of analytical grade and highest purity.

### Experimental design

The animals were equally allocated into five groups of ten rats each. The C group (control group) was injected intraperitoneally (*ip*) daily with 1 mL physiological saline solution for 30 consecutive days. The S group (sham or vehicle group) was *ip* injected daily for 30 days with physiological saline plus absolute ethanol with a final concentration below 0.1%. The TXL-treated group received *ip* injections of 7.5 mg/kg bw TXL once a week (days 0, 7, 14, 21, and 28) at 11 am. This dosage was selected based on previous studies (6,12). The MLT-treated group received a daily *ip* injection of 10 mg/kg bw MLT for 30 days at 8 am. MLT was freshly dissolved in a minimum volume of absolute ethanol (0.5 mL) and diluted with physiological saline to the desired concentration (10 mg/kg bw). The final concentration of ethanol was below 0.1%. The dose and preparation of MLT were based on previous studies (13,14). The MLT+TXL-treated group was treated with MLT (10 mg/kg bw, daily) and TXL (7.5 mg/kg bw, once a week) concomitant in the same manner.

At the end of the experimental period, the rats were anesthetized using diethyl ether, dissected, and their testes were carefully excised, washed with physiological saline, and processed for histological, histomorphometrical, immunohistochemical, and ultrastructural investigations.

### Histological preparation

Samples of the testes were cut into small segments and fixed for 24 h in aqueous Bouin's fixative. Then, samples were prepared by routine protocols of paraffin sectioning as previously described (15). Ehrlich's hematoxylin and eosin (HE)-stained sections were examined and photographed using a BX-40 Olympus (Japan) compound light microscope provided with a Panasonic CD-220 camera (China).

### Histomorphometrical measurements

Five random fields from HE-stained testicular cross-sections of control and experimental groups were selected and analyzed using a computed image analysis system (Leica Qwin, 500 Software, Germany). The thickness of the tunica albuginea, diameter of seminiferous tubules, and height of germinal epithelia were measured, and the numbers of seminiferous tubules without sperm, apoptotic germinal epithelia, and apoptotic Leydig cells were counted.

### Immunohistochemical preparations

Buffered neutral formalin-fixed paraffin-embedded testicular tissue sections from the control and treated rats were prepared and immunostained for detection of B-cell lymphoma 2 (Bcl-2), P53, and caspase3 (Cas3) reactive proteins following the standard avidin-biotin complex (ABC) method previously described (16). The antibodies and all reagents were used in conformity with the manufacturer's guidelines and recommendations. The antibody panel used is outlined in Table 1. Briefly, 5-μm-thick sections were deparaffinized, rehydrated, and rinsed in phosphate-buffered saline (PBS) for 10 min. Endogenous peroxidase activity was blocked with 3% hydrogen peroxide. Subsequently, the sections were incubated for 1-2 h at room temperature with the appropriate dilution of the primary antibodies and refrigerated overnight at 4°C. Then, they were washed in PBS 5 times, incubated with biotinylated goat anti-polyvalent for 10 min, followed by incubation with ABC for 1 h. Next, the sections were washed in PBS, and then incubated in diaminobenzidine tetrahydrochloride (pH 7.2) with 10 mL H_2_O_2_ for 7-9 min followed by 4 changes of PBS. Sections were then counterstained with Mayer's hematoxylin for 2 min, rinsed with tap water, dehydrated, cleared, and covered by cover slips. In each case, negative controls were stained by the routine immunostaining sequence, but the primary antibody was omitted and replaced by PBS.

**Table 1:** Antibodies used for immunohistochemical investigation.

Antibody	Code	Clone	Antigen retrieval	Dilution	Sources	Supplier
Bcl-2	MA5-11757	100/D5	PBS, pH 7.4 with 0.2% BSA	1:50	Mouse/IgG1, kappa	Thermo Fisher Scientific (USA)
P53	MA5-12557	DO-7	PBS, pH 7.4	1:100-1:200	Mouse/IgG2b, kappa	Thermo Fisher Scientific
Cas3	MA5-11516	3CSP01 (7.1.44)	PBS, pH 7.4 with 0.2% BSA	1:50-1:100	Mouse/IgG2a	Thermo Fisher Scientific

### Quantification of immunohistochemical parameters

Image analysis was used to quantify the immunoreactivity of Bcl-2, P53, and Cas3 reactive proteins. The immunostained sections were first examined and photographed by a compound light microscope equipped with a computed image analysis system (Leica Qwin, 500 Software) to assess the number of positive cases and the location of immunostaining within the tissue cells. Cells were considered to be positive if membrane and/or cytoplasm were stained brown. Bcl-2, P53, and Cas3 immunoreactivities were assessed by estimating the area percentage of the positive immunostained cells in relation to the total number of cells examined at ×200 magnification in 5 selected fields with a standard measuring frame of 11,434.9 mm^2^ using the computed image analysis system at Department of Oral and Dental Pathology, Faculty of Dental Medicine for Girls, Al-Azhar University. The image analyzer was first calibrated automatically to convert the measurement units (pixels) produced by the image analyzer program into actual micrometer units. Mean percentages of immunoreactive cells were obtained for all specimens in each group.

### Ultrastructural preparation

Small segments of the freshly excised testes were instantaneously fixed for 24 h in cold 4F1G (4% formalin and 1% glutaraldehyde, pH 2.2), then post-fixed for 2-4 h in 1% phosphate buffered osmium tetroxide (pH 7.3). After fixation, specimens were processed following the previously described technique (17). The stained grids were carefully examined and photographed by JEOL.JEM-1200-EX-electron microscope (Japan) equipped with a camera at the Central Laboratory of Faculty of Agriculture, Cairo University.

### Statistical analysis

Data are reported as means±SE. Differences in animal groups were compared by one-way analysis of variance (ANOVA) using SPSS/17.0 software (IBM, USA). Statistical significances among groups were assessed by Tukey's multiple comparison *post hoc* test and a P value ≤0.05 was considered statistically significant.

## Results

### Histological results

**Figure 1:**
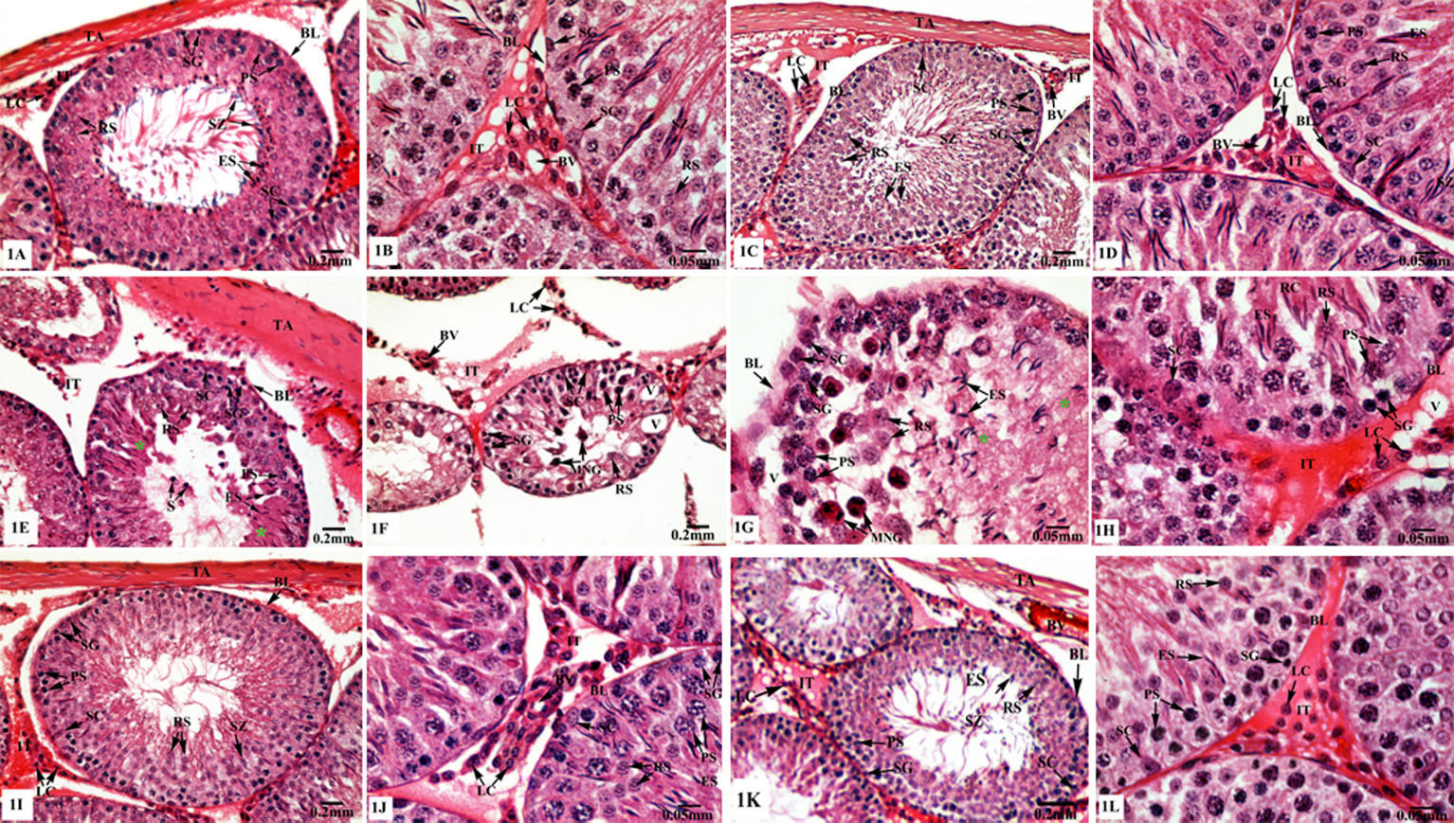
Photomicrographs of the testes of control and experimental animal groups stained with HE. **A** and **B**, control group with normal tunica albuginea (TA) and seminiferous tubules having basal laminae (BL), Sertoli cells (SC), spermatogonia (SG), primary spermatocytes (PS), rounded (RS) and elongated (ES) spermatids and spermatozoa (SZ), as well as interstitial tissues (IT) with Leydig cells (LC). **C** and **D**, sham group with intact testicular architecture. **E**-**H**, taxol (TXL)-treated rats with thickened tunica albuginea (TA) surrounding deteriorated tubules having ruptured basal laminae (BL), distorted SC, and apoptotic germinal cells; SG, PS, RS, and ES with residual cytoplasmic remnants (green asterisks), as well as vacuolation (V), sloughed germ cells (S), and multinucleated giant cells (MNG). Also, destructed interstitial tissues (IT) with pyknotic LC and congested blood vessels (BV) are displayed. **I** and **J**, melatonin (MLT)-treated rats with normal TA enclosing tubules having regular SC, SG, PS, RS, and ES, besides the IT with LC. **K** and **L**, regular testicular architecture of TA, seminiferous tubules with germinal epithelia and IT in MLT+TXL-treated rats. A, C, E, F, I, K: scale bar 0.2 mm. B, D, G, H, J, L: scale bar 0.05 mm.

Testes of control rats (Figure 1A and B) and of sham rats (Figure 1C and D) showed normal histological structure of active mature functioning seminiferous tubules associated with complete spermatogenic series and separated by regular interstitial tissues, having distinct Leydig cells and blood vessels. On the contrary, testes of TXL-treated rats revealed severe histopathological alterations as shown in Figure 1E-H. The tunica albuginea appeared thickened, and the seminiferous tubules exhibited irregular and ruptured basal laminae and conspicuous destruction of germinal epithelia that did not show any ordered progression of spermatogenic cells with dilated intercellular spaces between cells. Malformed Sertoli cells appeared separated from the basal laminae and very close to each other. Deteriorated tubules with apoptotic spermatogenic cells, vacuolation, and multinucleated giant cells, as well as sloughed germ cells were seen. Effects were more intense in spermatid differentiation, since there was a reduction in elongated spermatids, which appeared with abnormal amounts of residual casts of exfoliated cytoplasm that reflected on the formed spermatozoa, which appeared damaged and in lower numbers. Furthermore, the interstitial tissue showed congested blood vessels, vacuolation, and apoptotic Leydig cells. Testicular sections from MLT-treated rats (Figure 1I and J) had histological architecture similar to those of the control and sham groups. On the other hand, MLT+TXL-treated rats had obvious improvement of testicular structures, showing a nearly normal outermost layer of tunica albuginea and integral seminiferous tubules lined by intact basal laminae and composed of normal germinal cells and Sertoli cells, as well as intact interstitial tissue, which had regular clusters of Leydig cells as clearly seen in Figure 1K and L.

### Histomorphometrical analysis

As shown in Table 2, TXL-treated rats showed a significant rise (P≤0.05) in the thickness of tunica albuginea, while seminiferous tubule diameters and germinal epithelial heights were significantly reduced (P≤0.05) compared with those of control and sham groups. Also, there were significantly higher (P≤0.05) numbers of seminiferous tubules without sperm, apoptotic germinal epithelia, and apoptotic Leydig cells in the testicular tissue of TXL-exposed rats compared with those of control and sham groups. On the other hand, MLT supplementation into TXL-treated rats improved these impaired testicular histomorphometrical parameters of TXL intoxication. As also shown in Table 2, there was a significant decline (P≤0.05) in the thickness of tunica albuginea, while there were significant increases (P≤0.05) in the diameter of tubules and the height of germinal epithelia. Additionally, there were significantly lower (P≤0.05) numbers of tubules without sperm, apoptotic germinal epithelia, and apoptotic Leydig cells in the MLT+TXL group compared with those of TXL-treated rats. MLT-treated rats did not exhibit any significant alteration in these parameters except for the thickness of tunica albuginea, which had a significant increase (P≤0.05) compared to control and sham groups.

**Table 2:** Histomorphometrical analysis of testicular parameters of control and experimental animal groups.

Testicular parameters	Animal groups				
	C	S	TXL	MLT	MLT+TXL
Thickness of tunica albuginea (μm)	31.4±1.69^a^	29.8±1.36^a^	55.4±8.96^b^	46±2.92^c^	33.4±1.99^a^
Diameter of seminiferous tubules (μm)	357.8±12.48^a^	356.4±16.86^a^	229.0±5.85^b^	354.8±9.89^a^	340.4±7.30^a^
Germinal epithelium height (μm)	84.0±2.55^a^	83.6±4.23^a^	54.2±1.43^b^	83.4±3.01^a^	79.4±1.83^a^
Number of seminiferous tubules without sperm	1.0±0.32^a^	1.2±0.37^a^	12.2±0.86^b^	2.0±0.55^a^	2.2±0.37^a^
Number of apoptotic germinal epithelia	9.2±1.11^a^	9.6±0.51^a^	60.6±1.91^b^	13.4±0.81^a^	28.8±1.77^c^
Number of apoptotic Leydig cells	1.2±0.37^a^	1.2±0.37^a^	4.4±0.68^b^	1.4±0.51^a^	1.0±0.32^a^

Data are reported as means±SE (n=5 in each group). Means with different superscript letters in the same row differ significantly at 5% (P≤0.05, ANOVA). C: control; S: sham; TXL: taxol; MLT: melatonin.

### Immunohistochemical results

#### Bcl-2 immunoreactivity

**Figure 2:**
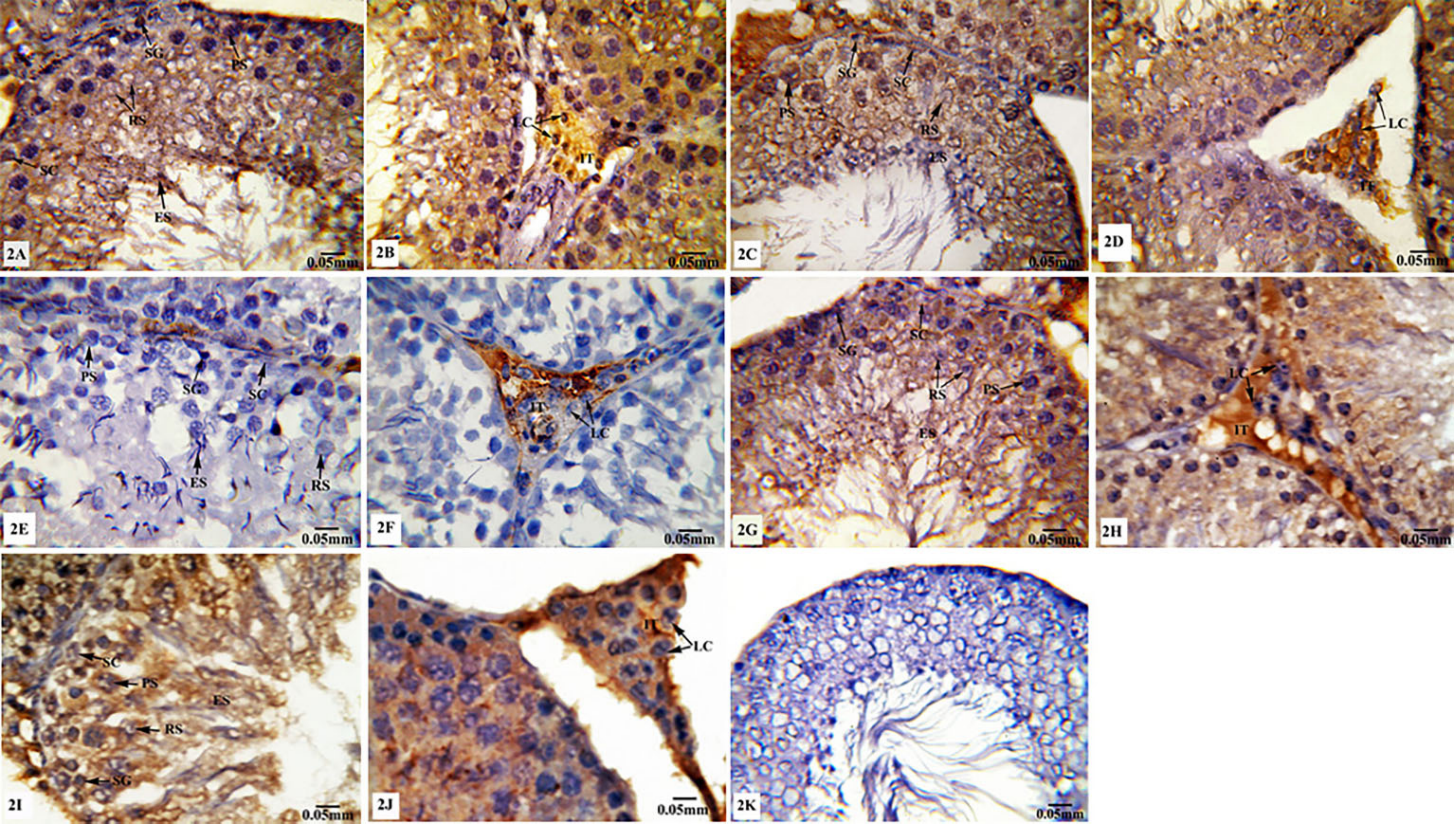
Photomicrographs of testicular tissues of control and experimental animal groups stained for Bcl-2 immunoreactivity. **A** and **B**, control group; **C** and **D**, sham group; **E** and **F**, taxol (TXL)-treated group; **G** and **H**, melatonin (MLT)-treated group; **I** and **J**, MLT+TXL-treated group. There was no staining on the negative control sample (**K**). SC: Sertoli cells; SG: spermatogonia; PS: primary spermatocytes; RS: rounded spermatids; ES: elongated spermatids; LC: Leydig cells; IT: interstitium. Scale bar: 0.05 mm.

Testicular tissues from TXL-treated rats (Figure 2E and F) revealed negative Bcl-2 immunoreactivity in germinal and Sertoli cells, and moderate positive immunostaining in Leydig cells compared with those of the control (Figure 2A and B) and sham (Figure 2C and D) groups. However, treatment of animals with MLT concomitant with TXL up-regulated the immunoexpression of Bcl-2 in spermatogenic, Sertoli, and Leydig cells (Figure 2I and J). Testicular sections from MLT-treated rats (Figure 2G and H) showed similar Bcl-2 immunoreactivity compared with those of the control and sham groups. No staining was observed of sections from the negative control (Figure 2K). As revealed in Table 3, a significant decrease (P≤0.05) in the immunohistochemical quantitative value of Bcl-2 expression was observed in TXL-treated rats, which was significantly modulated by MLT supplementation. Meanwhile, MLT-treated rats did not display any significant alteration in the value of Bcl-2 immunoexpression compared to control and sham groups.

**Table 3:** Percentage of cells immunostained for Bcl-2, P53, and Cas3 reactive proteins in testicular tissues of control and experimental animal groups.

Immunohistochemical parameters	Animal groups				
	C	S	TXL	MLT	MLT+TXL
Bcl-2	38.28±1.94^a^	36.26±1.93^a^	7.66±0.78^b^	35.83±2.32^a^	33.30±0.85^a^
P53	11.34±1.68^a^	12.00±1.67^a^	47.18±2.23^b^	12.32±1.17^a^	18.43±2.01^a^
Cas3	20.15±1.27^a^	20.7±1.00^a^	52.62±2.45^b^	21.89±1.33^a^	30.21±1.90^a^

Data are reported as means±SE (n=5 in each group). Means with different superscript letters in the same row differ significantly at 5% (P≤0.05, according to ANOVA). C: control; S: sham; TXL: taxol; MLT: melatonin.

#### P53 immunoreactivity

**Figure 3:**
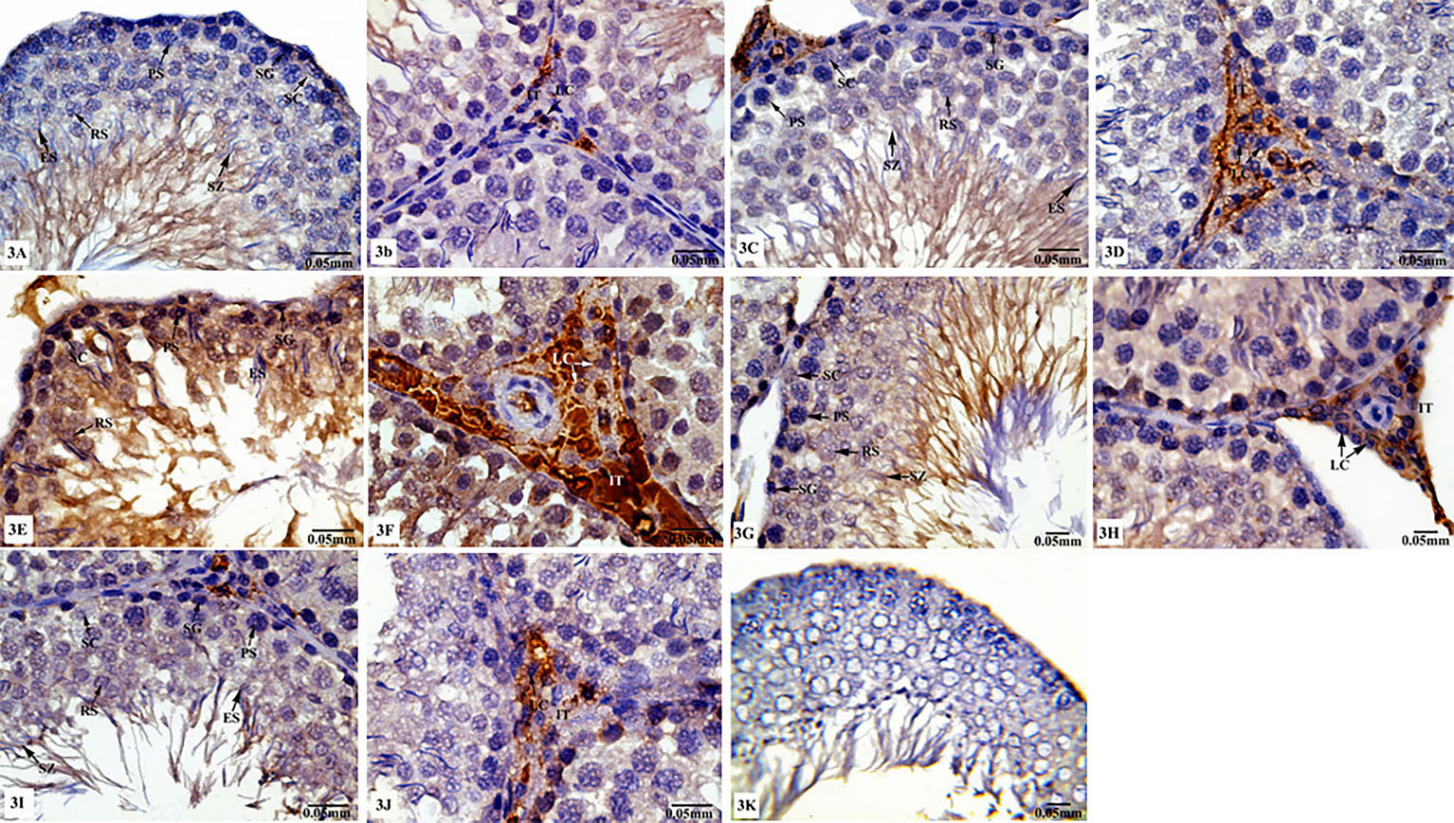
Photomicrographs of testicular tissues of control and experimental animal groups stained for P53 immunoreactivity. **A** and **B**, control group; **C** and **D**, sham group; **E** and **F**, taxol (TXL)-treated group; **G** and **H**, melatonin (MLT)-treated group; **I** and **J**, MLT+TXL-treated rats. There was no staining on the negative control sample (**K**). SC: Sertoli cells; SG: spermatogonia; PS: primary spermatocytes; RS: rounded spermatids; ES: elongated spermatids; LC: Leydig cells; IT: interstitium. Scale bar: 0.05 mm.

Cross-sections of control (Figure 3A and B) and sham (Figure 3C and D) rats revealed negative immunoreactivity for P53 in spermatogenic and Sertoli cells, whereas weak immunostaining was seen in Leydig cells. On the contrary, testicular tissues from rats treated with TXL showed increased P53 immunoreactivity in germinal, Sertoli, and Leydig cells as shown in Figure 3E and F. However, animals treated with MLT+TXL showed marked decrease of P53 immunoreactivity (Figure 3I and J). MLT-treated rats (Figure 3G and H) showed similar P53 immunostaining to those of control and sham groups. Under this condition, there was no staining of sections from the negative control as shown in Figure 3K. Table 3 shows that MLT-exposed rats did not have a significant variation in P53 immunoexpression compared with the control and sham groups, whereas a significant increase (P≤0.05) in the value of P53 immunoexpression was recorded in TXL-treated group. Furthermore, MLT+TXL treated animals showed a significant decrease (P≤0.05) in P53 immunoexpression compared to the animals treated with TXL alone.

#### Cas3 immunoreactivity

**Figure 4:**
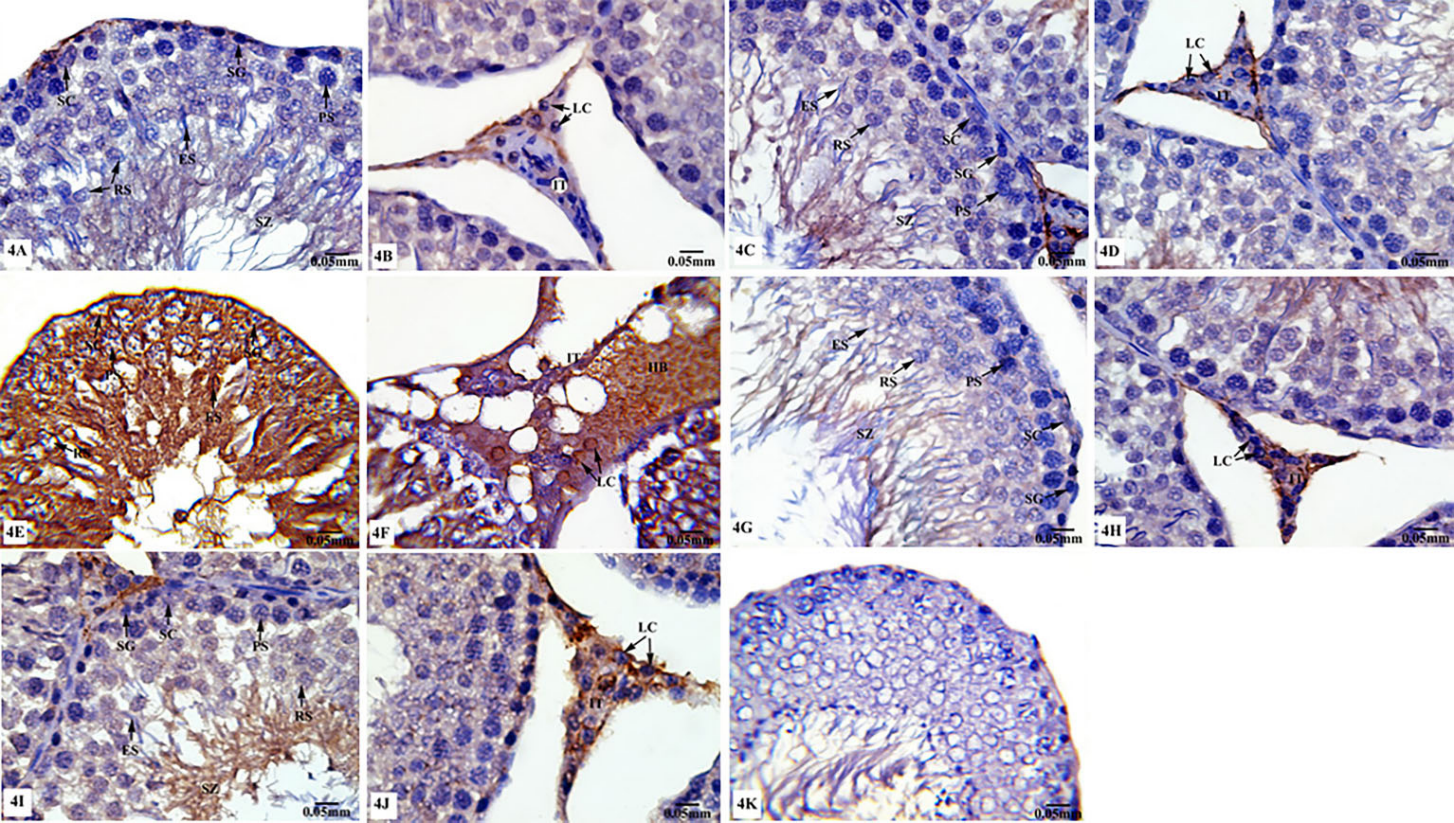
Photomicrographs of testicular tissues of control and experimental animal groups stained for Cas3 immunoreactivity. **A** and **B**, control group; **C** and **D**, sham group; **E** and **F**, taxol (TXL)-treated group; **G** and **H**, melatonin (MLT)-treated group; **I** and **J**, MLT+TXL-treated group. There was no staining on the negative control sample (**K**). SC: Sertoli cells; SG: spermatogonia; PS: primary spermatocytes; RS: rounded spermatids; ES: elongated spermatids; LC: Leydig cells; IT: interstitium. Scale bar: 0.05 mm.

Immunohistochemical analysis of testicular sections of control (Figure 4A and B) and sham rats (Figure 4C and D) showed negative reactivity for Cas3 immunostaining in spermatogenic and Sertoli cells, while mild positive Cas3 immunoreaction was detected in the interstitial tissues. Testicular sections of TXL-treated rats showed strong positive staining for Cas3 in germinal epithelia and interstitial tissue as clearly seen in Figure 4E and F. Testes sections of rats from the MLT group (Figure 4G and H) revealed similar Cas3 immunoreactivity to control and sham groups. Also, MLT co-administered with TXL showed similar Cas3 immunostaining (Figure 4I and J) to those of control and sham groups. As seen in Figure 4K, there was no staining on the negative control sample. According to Table 3, testes sections from TXL treated-rats evoked a significant increase (P≤0.05) in the value of Cas3 immunoexpression compared to control and sham rats and tissues of rats treated with MLT did not show any significant change in Cas3 immunoexpression values compared to the control group. On the other hand, MLT+TXL resulted in modulation of Cas3 immunoexpression value compared with the rats subjected to TXL only.

### Ultrastructural results

**Figure 5:**
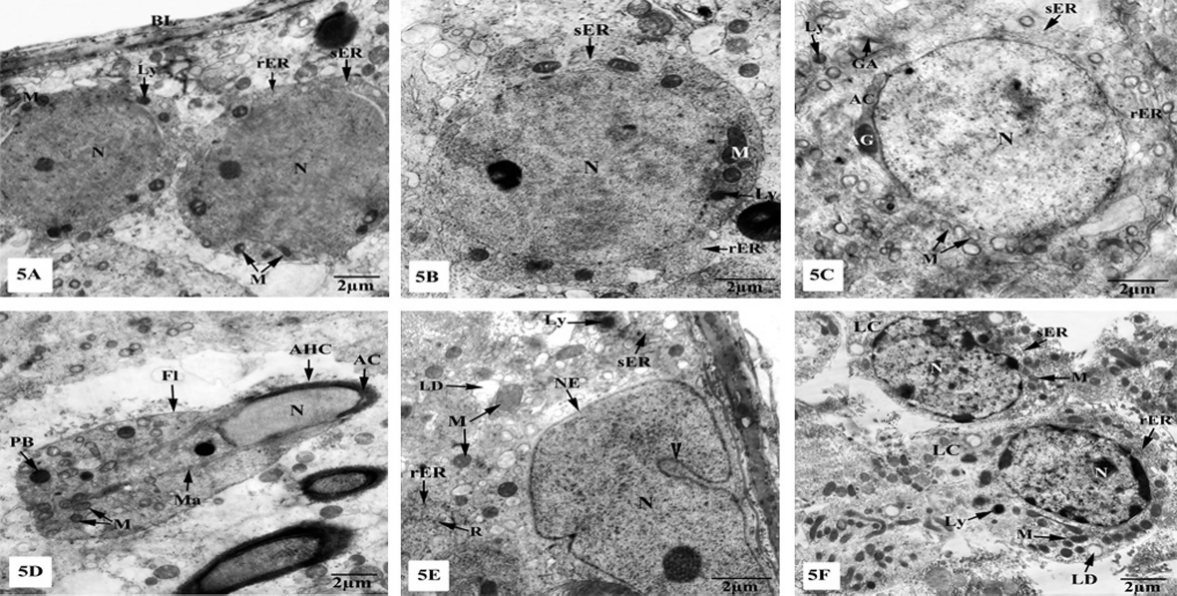
Electron micrographs of the testes of control and sham groups showing (**A**) spermatogonia resting upon a thin basal lamina (BL) and possess nuclei (N), mitochondria (M), lysosomes (Ly), rough endoplasmic reticulum (rER), and smooth endoplasmic reticulum (sER); (**B**) primary spermatocyte with N, M, Ly, sER, and rER; (**C**) rounded spermatid with vacuolated M, Golgi apparatus (GA), Ly, rER, and sER, as well as acrosomal granule (AG) and acrosomal cap (AC), which cover the nucleus; (**D**) elongated spermatid showing N covered with AC forming the acrosomal head cap (AHC), the manchette (Ma), and the flagellum (Fl), with M and phagosomal bodies (PB); (**E**) Sertoli cell appeared with irregular N, enclosed within a nuclear envelope (NE) with a deep indentation (▸), and its cytoplasm containing M, Ly, lipid droplets (LD), free ribosomes (R), sER, and rER; (**F**) interstitial tissue revealing Leydig cells (LC) with numerous M, sER, rER, Ly, LD, and N. Scale bar 2 μm.

Specimens of testes from control and sham groups showed normal structural architecture of the seminiferous tubules and interstitial tissues as illustrated in Figure 5A-F. Spermatogonia lie on a thin basal lamina and had slight stacks of rough endoplasmic reticulum (rER), scattered mitochondria, lysosomes, and intact nuclei (Figure 5A). Primary spermatocytes appeared large, spherical in shape, and containing rounded nuclei and granular cytoplasm that had mitochondria and both types of endoplasmic reticula (Figure 5B). Rounded spermatids appeared ovoid in shape with oval nuclei and cytoplasm containing slight stacks of rER, vacuolated mitochondria, lysosomes, and well-developed Golgi apparatus forming acrosomal granules and caps that attached to the anterior poles of the nuclei (Figure 5C). Elongated spermatids had elongated nuclei covered with the acrosomal caps forming the acrosomal head caps, and manchette laterally limiting the nuclei and extending into the flagella that mainly contained numerous mitochondria (Figure 5D). Sertoli cells possessed irregular nuclei containing prominent nucleoli, homogeneous chromatin material, and nuclear envelopes that exhibited deep indentations. The cytoplasm was distinguished by the existence of mitochondria, cisternae of sER and rER, free ribosomes, lysosomes, and lipid droplets (Figure 5E). The interstitial tissues appeared with prominent Leydig cells being oval in shape with obvious nuclei, and their cytoplasm contained sER, rER, mitochondria, lysosomes, and lipid droplets (Figure 5F).

**Figure 6:**
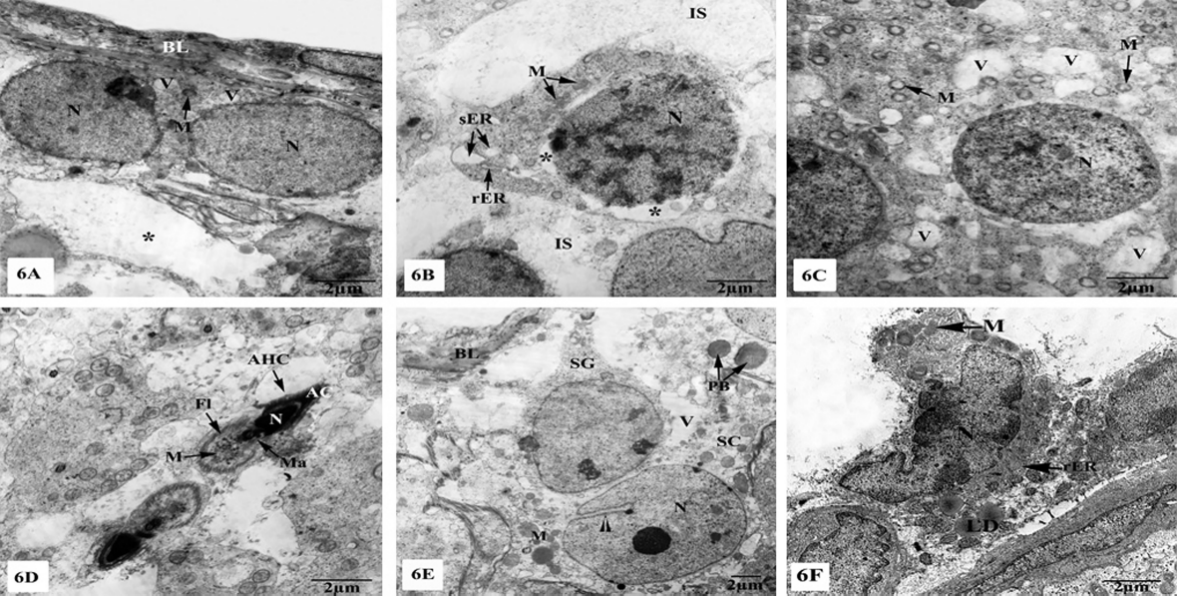
Electron micrographs of the testes of taxol (TXL)-treated rats showing (**A**) deteriorated spermatogonia resting upon thickened basal lamina (BL) and have few electron-dense mitochondria (M), cytoplasmic vacuoles (V), and marked dilatations of intercellular spaces (asterisks); (**B**) atrophied primary spermatocyte with karyorrhexed nucleus (N) separated from the cytoplasm leaving empty zones (asterisks), few electron-dense M, fragmented rough endoplasmic reticulum (rER), and dilated smooth endoplasmic reticulum (sER), as well as widened intercellular spaces (IS); (**C**) degenerated rounded spermatids appeared with karyorrhexed N and cytoplasm with few M and large V; (**D**) atrophied elongated spermatids having distorted acrosomal head caps (AHC), electron-dense N, condensed acrosomes (AC), electron-dense small manchettes (Ma), and short flagella (Fl) with disarranged electron-dense M; (**E**) deformed Sertoli cell separated from the basal lamina (BL) and has irregular N with deep nuclear indentation (▸), electron-dense M, phagosomal bodies (PB) and V. Club-shaped spermatogonium (SG) is also markedly detected; (**F**) apoptotic Leydig cell revealing highly irregular and fragmented N and degenerated cytoplasm having lipid droplets (LD), electron-dense M, and fragmented rER. Scale bar 2 μm.

The testes of TXL-treated rats showed remarkable ultrastructural alterations in the seminiferous tubules and interstitial tissues as seen in Figure 6A-F. The basal laminae appeared thickened and irregular (Figure 6A and E). Spermatogonia appeared small in size, irregular or club shape, having few electron-dense mitochondria and cytoplasmic vacuoles. Therefore, the intercellular spaces between these deformed spermatogonia appeared markedly dilated (Figure 6A and E). Primary spermatocytes appeared also small in size, revealing rather karyorrhexed nuclei that separated from the surrounding cytoplasm leaving empty spaces. In addition, few electron-dense mitochondria, fragmented stacks of rER, and dilated cisternae of sER were seen (Figure 6B). Rounded spermatids appeared with ruptured plasma membranes, karyorrhexed nuclei, few mitochondria, and increased cytoplasmic vacuoles as shown in Figure 6C. Atrophied elongated spermatids had electron-dense acrosomal head caps with nuclei exhibiting pyknosis, and acrosomes were discontinuous. The manchette appeared as a patch aligned to the nuclei on one side, and their flagella was short having disarranged and intense electron-dense mitochondria (Figure 6D). Sertoli cells appeared markedly detached from the basal lamina and with irregular nuclei, electron-dense mitochondria, vacuoles, and phagosomal bodies (Figure 6E). The interstitial tissues from TXL-treated testes revealed obvious apoptotic Leydig cells, with highly irregular and fragmented nuclei, electron-dense mitochondria, fragmented rER, and lipid droplets (Figure 6F).

**Figure 7:**
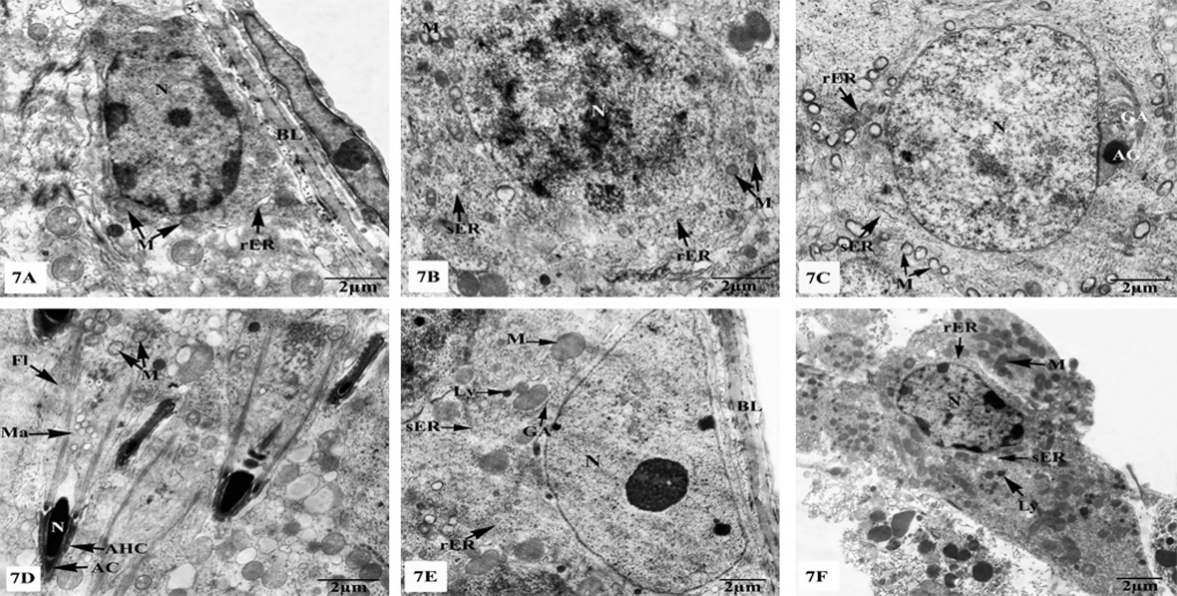
Electron micrographs of the testes of melatonin (MLT)-treated rats showing (**A**) intact spermatogonium resting on basal lamina (BL) having oval nucleus (N), mitochondria (M), and rough endoplasmic reticulum (rER); (**B**) primary spermatocyte with N and cytoplasmic organelles including M, rER, and smooth endoplasmic reticulum (sER); (**C**) fully-developed rounded spermatid with N, acrosomal granule (AG), Golgi apparatus (GA), vacuolated M, sER, and rER; (**D**) elongated spermatids displaying elongated N, acrosomal caps (AC), acrosomal head caps (AHC), manchettes (Ma), and flagella (Fl), with M. (**E**) Sertoli cell resting upon a thin basal lamina (BL) and has well developed M, sER, rER, GA, and N; (**F**) intact Leydig cell with N, rER, sER, M, and lysosomes (Ly). Scale bar 2 μm.

**Figure 8:**
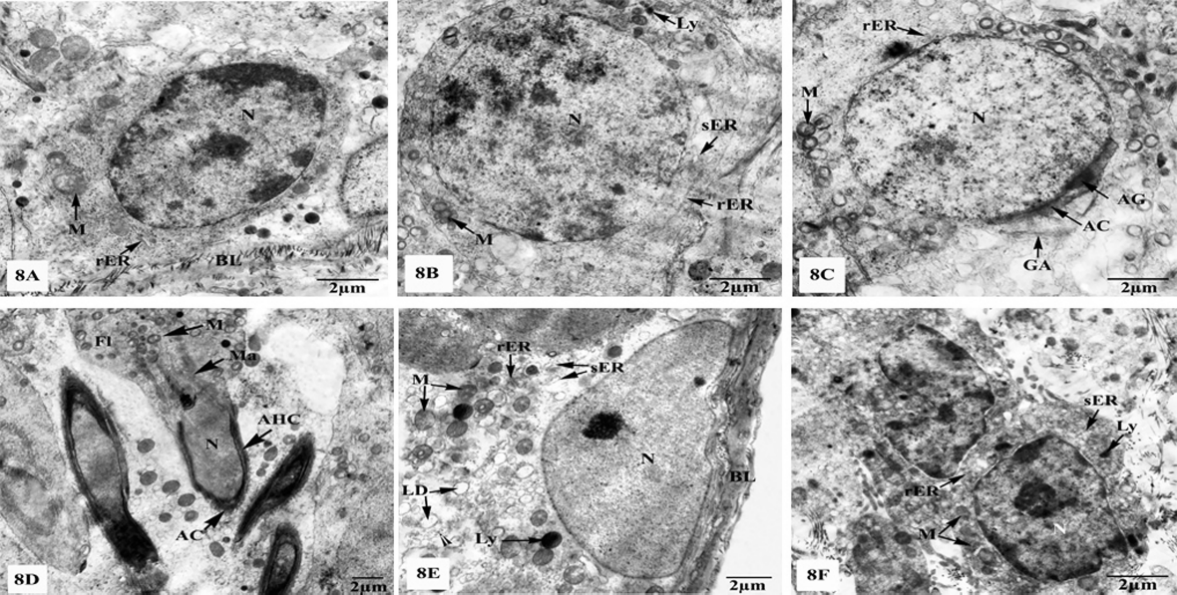
Electron micrographs of the testes of melatonin+taxol (MLT+TXL)-treated rats showing (**A**) nearly normal spermatogonium resting upon basal lamina (BL), and possesses oval nucleus (N), mitochondria (M), and rough endoplasmic reticulum (rER); (**B**) intact primary spermatocyte with N, M, rER, smooth endoplasmic reticulum (sER), and lysosomes (Ly); (**C**) regular rounded spermatid with intact N, acrosomal granule (AG), acrosomal cap (AC), Golgi apparatus (GA), vacuolated M, and rER; (**D**) nearly normal elongated spermatids with regular acrosomal head caps (AHC), N, AC, manchettes (Ma), flagella (Fl), and M; (**E**) well-developed Sertoli cell laying on a thin BL with N, rER, sER, M, free ribosomes (▸), and lipid droplets (LD); (**F**) interstitium with Leydig cells appeared with prominent N and cytoplasmic organelles including M, rER, sER, and Ly. Scale bar 2 μm.

Both seminiferous tubules and the interstitial tissues of the MLT-treated group exhibited similar ultrastructural features to the control group as clearly shown in Figure 7A-F. Similarly, the testes of MLT+TXL-treated group revealed marked improvement of the fine structural characteristics of the spermatogenic and Sertoli cells, as well as the interstitial Leydig cells as clearly observed in Figure 8A-F.

## Discussion

The findings of the present investigation disclosed a myriad of histological and ultrastructural changes in the testicular tissues of TXL-treated rats, including irregularity and thickening of tunica albuginea and basal laminae, as well as degenerative alterations in spermatogenic, Sertoli, and Leydig cells. These harmful effects following TXL intoxication were also observed in the histomorphometrical analysis of testicular parameters of treated rats.

Various testicular disorders are correlated with the thickening of the seminiferous tubular wall, which disrupts the connection between the interstitium and the inner tubular components. The basal lamina plays a major role in maintaining the transport of substances like oxygen, nutrients, metabolites, and hormones between the interstitium and the spermatogenic epithelium, and in maintaining structural and functional completeness of the testicular tissue, which is vital for maintaining the normal process of spermatogenesis (18). The key element of the mammalian basal lamina is subtype IV collagen, which is generated by Sertoli and myoid cells (19). The results of previous studies confirm that overexpression of subtype IV collagen is linked to thickening of the basement membranes, which coincides with spermatogenic impairment in humans and other animals (20).

Furthermore, Sertoli cells distortion was apparent in the testes of TXL-treated rats. As a consequence of Sertoli cells disturbance, widened intercellular spaces and lack of contact between spermatogenic cells emerge. This triggers germ cell loss, leading to testicular tissue destruction and lastly, infertility (21). Sertoli cells stimulate the production of germ cells and retain their viability through the secretion of nutrients and hormones into the blood-testis barrier (22). Also, Sertoli cells possess a significant role in the conservation of spermatogenesis by controlling spermatogenic cells in the successive stages of mitosis, meiosis, and differentiation (23). Furthermore, the impaired Sertoli cells trigger alterations or reduction of the tubular secretory substances, causing sloughing or shedding of germ cells, besides spermatogenic cell death (24). On the other hand, Ghasemi et al. (13) reported that the cytotoxic agents target spermatogenic cells rather than Sertoli and Leydig cells because of their high mitotic activity.

Additionally, multinucleated giant cells in the testes of TXL-exposed rats could be related to a particular form of degenerated spermatogenic cells, which resulted from disruption of Sertoli cell conservation or disruption of cytoplasmic bridges between spermatocytes or spermatids. This impairment might cause the contents of conjoined cells to fuse and eventually leads to apoptosis due to cell differentiation inability (25). Furthermore, exfoliation of damaged spermatogenic cells forming cellular casts in tubular lumens was detected in TXL-treated testes. Sloughing of germ cells might be linked to the degeneration of these cells, which could result from perturbation of membrane structures, triggering their shedding in the lumens of the tubules and forming cellular casts.

Moreover, most germinal epithelia showed vacuolation. These vacuoles could be regarded as an apoptotic cell feature (22). During normal spermatogenesis, testicular cell apoptosis is crucial for maintaining the right proportion between Sertoli cells and gametes. Meanwhile, different pathological circumstances such as hormonal depletion, toxic materials, drugs, heat stress, and ionizing radiation exposure may significantly increase germ cell apoptosis (26).

Leydig cells were also affected by TXL treatment, showing a significant decrease in their numbers and size and increased lipid droplets inside their cytoplasm. Such defects in Leydig cells may be linked to the intimate relationship between them and their vasculature, which place them at risk for any exogenous toxin causing spermatogenesis arrest owing to the decrease of testosterone secretion (27).

Mitochondrial degeneration was one of the most significant ultrastructural changes observed in nearly all spermatogenic, Sertoli, and Leydig cells after treatment with TXL. Such mitochondrial degeneration could be due to the reactive oxygen species (ROS) formation that adversely affects the antioxidant system after TXL therapy, as previously reported (28). The imbalance between ROS formation and removal can cause oxidative stress in tissues (29). This oxidative stress generated by TXL can result in the deterioration of testicular structures and functions, as well as influence sperm morphology, motility, and count.

The immunohistochemical results showed that TXL administration induced testicular apoptosis through decreased anti-apoptotic protein Bcl-2 and increased apoptotic tumor suppressor protein P53 immunoexpressions. Bcl-2 is an apoptosis regulatory gene, which contributes to tumorigenesis by inhibiting programmed cell death and promoting cell survival. It is localized mostly in the outer membrane of mitochondria, and at a minimum amount in the nuclear and endoplasmic reticula membranes. It is known to regulate cell death by controlling the mitochondrial membrane permeability (30), whereas P53 acts as a nuclear transcription agent that transactivates genes implicated in regulation or improvement of cell cycle, apoptosis, and genomic stability by means of various mechanisms. P53 is found inside the nucleus and has a key role in controlling cell division and cell death. Additional activities of P53 were seen in the cytoplasm, also inducing apoptosis and inhibiting autophagy (31).

A significant increase of the immunoexpression level of Cas3 was observed in the testicular tissues of TXL-treated rats. Cas3 is a protein that has a vital role in both the death receptor and the mitochondrial pathway (extrinsic and intrinsic apoptosis, respectively). The activation of the Cas family members results in the loss of cell structure and function, causing cell apoptosis. Moreover, since Cas3 exerts irreversible damages, the caspase-inhibiting characteristics of Bcl-2 should not be neglected. Bcl-2 displays anti-apoptotic activity via pro-caspase sequestration and caspase self-cleavage inhibition (32).

Several mechanisms underlying the induction of apoptosis by TXL have been explained and shown to depend on cellular context and concentration. The direct connection of TXL to the microtubulin beta subunit instantly resulted in the arrest of the G2/M cell cycle followed by apoptotic cell death, which is considered the basis for TXL cytotoxicity (33). Bcl-2 phosphorylation is the hallmark of TXL-induced cell death, although the association between this phenomenon, mitotic arrest, and apoptosis is still controversial. A stronger suppression of apoptosis by Bcl-2 has also been reported previously (34). Furthermore, TXL-triggered cell death had been demonstrated in the non-small cell lung cancer cell line NCI-H460^1^, primarily via Cas-independent pathways (35).

TXL has also been shown to increase oxidative stress in cancer cells. Aggregation of ROS is an early and pragmatic step in TXL-induced apoptosis of cancer cells. This oxidative stress induced by TXL may also contribute to its toxic effects against non-targeted tissues (28). Furthermore, oxidative stress is one of the key factors leading to apoptosis of testicular cells. Both germinal cells and sperms are susceptible to such stress because they contain high levels of both long-chain and very long-chain highly unsaturated fatty acids, which are easily oxidized and lead to cellular impairment, posing a threat to male reproduction (36). Therefore, spermatogenesis must be protected from such oxidative stress during TXL chemotherapy protocols.

The current study investigated the probable protective effect of MLT supplementation at a dose of 10 mg/kg bw on the testicular tissues of rats intoxicated with TXL. The rats treated with both substances showed a marked improvement in the histological, histomorphometrical, and ultrastructural features of their testicular tissues compared to TXL-treated rats. Also, the current immunohistochemical results showed that MLT exhibited anti-apoptotic effects against testicular apoptosis induced by TXL, which was emphasized by the significant elevation in immunoexpression of active Bcl-2 protein and decrease in P53 and Cas3 immunoexpressions in the testicular tissues of the rats co-treated with MLT and TXL.

Consistent with the present results, MLT protected testicular tissues in animal models of testicular injury triggered by different anti-cancer drugs (37). One of the most interesting properties of MLT is its antioxidant ability, which promotes its use as an adjuvant to radiotherapy and chemotherapy or as a component of cancer treatment protocols with other anti-cancer activity molecules (38).

TXL-treated rats showed a significant increase in the thickness of tunica albuginea as a result of increased fibrosis, a consequence of cellular apoptosis induced by TXL. In contrast, MLT co-administered with TXL to rats modulated cellular apoptosis induced by TXL and subsequently restored the thickness of tunica albuginea. The anti-fibrotic property of MLT may also have contributed to this modulation of tunica albuginea thickness (39).

The protective role of MLT in protecting testes could be due to its antioxidant impact on both somatic and germ cells, and also to its influence on the hypothalamic-pituitary-gonadal axis. Another power of MLT is its amphiphilic property. Unlike other antioxidants, which are either lipophilic or hydrophilic, MLT was found to be able to cross physiological boundaries and reduce oxidative damage in both aqueous and lipid cellular environments. Also, MLT has been reported to have both direct and indirect antioxidant properties. The direct impact of MLT was observed in the male reproductive system and in testosterone synthesis from Leydig cells in some animals. This effect can be explained by the direct free radical scavenging and/or by the activation of DNA repair enzymes. On the other hand, MLT shows an indirect antioxidant effect by increasing the activities of glutathione peroxidase (GSH-Px) and superoxide dismutase (SOD) (40). Finally, understanding the mechanisms and properties of MLT can make an important contribution to the protection of male fertility in the clinical setting.

In conclusion, the current experimental study revealed that MLT supplementation protected the testicular tissues of rats exposed to TXL from its deleterious effects through the antioxidant and anti-apoptotic properties of MLT. Furthermore, this investigation revealed that MLT can be used as a protective agent during chemotherapeutic treatment to prevent damage to the testes.
